# Response to: Comment on: Relevance of Posterior Gastric vessel in Bariatric Surgery

**DOI:** 10.1007/s11695-021-05230-2

**Published:** 2021-07-24

**Authors:** Prakhar Gupta, Shivanshu Misra, S. Saravana Kumar, P. Praveen Raj

**Affiliations:** 1 Department of Upper GI surgery, GEM Hospital and Research Center, Coimbatore, India; 2 Department of Bariatric Surgery, GEM Hospital and Research Center, Coimbatore, Tamil Nadu India

First of all, we would like to thank you for your comments on the article “Relevance of Posterior Gastric Vessel in Bariatric Surgery.”

We certainly agree a pre-op study with CT angiography and a prospective assessment would have had better significance than a retrospective video review. However, we preferred to report our findings based on the fact that we already noted the presence of the vessel in a large percentage of cases even with this methodology, in relation to bariatric procedures, much higher than reported previously. Hence, this is only an anatomical observational study where we wanted to study the presence and relevance of posterior gastric artery in bariatric surgery. Due to the variable incidence and origin of this vessel, it has not been described consistently in the literature. Though its importance has been described for surgeries over the stomach and pancreas, until now, it has not been mentioned with respect to bariatric surgery.

During the most commonly performed bariatric surgeries, i.e., laparoscopic sleeve gastrectomy and laparoscopic Roux-en-Y gastric bypass, complete fundal mobilization is an important step, for both short-term and long-term successes. This vessel is usually encountered while performing posterior dissection near the fundus area. It is seen entering into the fundal region along with short gastric vessels. Its division is commonly required during sleeve gastrectomy for better fundal mobilization. While during gastric bypass division is only required in cases where it arises more medially, staying more medial to the vessel will ensure a good pouch.

The aim of this study was to assess the frequency and relevance of this vessel during bariatric procedures, as it has not been mentioned in bariatric literature before. Further anatomical studies based on CT angiography and intraoperative findings are warranted for delineating its course and frequency in bariatric practice.

